# Randomised controlled trial of mammographic screening in women from age 40: results of screening in the first 10 years

**DOI:** 10.1038/sj.bjc.6602396

**Published:** 2005-02-22

**Authors:** S Moss, I Thomas, A Evans, B Thomas, L Johns, (writing committee) for the Trial Management Group

**Affiliations:** 1 Cancer Screening Evaluation Unit, Institute of Cancer Research, Brookes Lawley Building, 15 Cotswold Road, Sutton, Surrey SM2 5NG, UK; 2 National Breast Screening Training Centre, City Hospital, Hucknall Road, Nottingham NG5 1PB, UK; 3 Jarvis Breast Screening, Diagnostic and National Training Centre, Stoughton Road, Guildford, Surrey GU1 1LJ, UK

**Keywords:** breast, screening, mammography, age, trial

## Abstract

Debate continues over the effectiveness of screening by mammography in women below age 50. We report here on results of screening in the first 10 years of a randomised trial to study the effect on breast cancer mortality of invitation to annual mammography from age 40 to 41 compared to first invitation to the 3-yearly UK national programme at age 50–52. The trial is taking place in 23 NHS breast screening centres. Between 1991 and 1997, 160 921 women were randomised in the ratio 1 : 2 to intervention and control arms. Screening is by two views at first screen and single view subsequently; data on screening up to and including round five are now complete. Uptake of invitation to screening is between 68 and 70% at all but the latest screening rounds. Rates of referral for assessment are 4.6% at first screen and 3.4% at subsequent screens. Invasive cancer detection rates are 0.09% at first screen, and similar at rescreens until the sixth and later screens. There is little evidence of regular mammography in the trial control arm. The setting of this trial within the NHS breast screening programme should ensure applicability of results to a national programme.

The effectiveness of screening for breast cancer by mammography in women below age 50 remains unproven, and is the subject of much debate. While evidence from randomised controlled trials (RCTs) increasingly suggests the existence of some benefit from screening in women below age 50 at trial entry, these trials were not specifically designed to address this question, and the extent to which the observed benefit results from screening in these women after they reach age 50 remains unclear. Analyses of some trials by age at diagnosis suggest a benefit from screening episodes under age 50, but such analyses reduce the comparability of the intervention and control arms and hence the reliability of the results.

The majority of population-based screening programmes that have been introduced offer screening to women from age 50, although in Sweden some counties invite women from age 40 (Shapiro *et al*, 1998). In the UK, the effectiveness of screening below age 50 was identified as one of the areas where further research was needed, at the time the national screening programme was introduced (Forrest, 1986). The Age Trial (International Standard Randomised Controlled Trial Number – ISRCTN: 24647151) was designed specifically to study the question of the benefit of starting screening from age 40. The unique features of this trial are annual mammography (frequently recommended for this age group) and freedom from bias due to screening episodes over age 50.

## MATERIALS AND METHODS

The design of the trial has been described in detail elsewhere (Moss, 1999). The aim was to randomise 195 000 women aged 40–41 in a ratio of 1 : 2 to an intervention arm offered annual screening by mammography, and a control arm with no intervention. Women in the intervention arm are invited for screening until the calendar year of their 48th birthday; after age 50, both they and women in the control arm are eligible for three-yearly invitation as part of the NHS Breast Screening Programme (NHSBSP), and will receive their first invitation between age 50 and 52 years. Screening in the trial is by two-view mammography at the first screen, with single view thereafter unless otherwise indicated. All women, including nonattenders, are reinvited annually unless they ask not to be contacted again. Women who move to areas not covered by the trial are not reinvited for screening as part of the trial, but are able to self-refer to either their previous or their nearest participating screening centre.

The sample size of the trial was calculated to give an 80% probability of demonstrating as statistically significant a reduction of 20% in breast cancer mortality at 10 years of follow-up at the 5% significance level, using a one-tailed test, on an ‘intention-to-treat’ basis. This was based on an estimated cumulative breast cancer mortality of 3.3 per 1000 women in the control group among an initially disease-free population, using the method described by Moss *et al* (1987).

The trial began in 1991, and includes 23 established NHSBSP centres(Table 1). Randomisation is individual, stratified by GP practice. Women were identified from the (then) Family Health Services Authority (FHSA) computerised register, as is the case for the national screening programme. Women were selected by year of birth to include those who would be 40 or 41 when offered their first invitation or who would become 40 in the calendar year of their first invitation. Prior Notification Lists (PNLs) were generated (before randomisation) and sent to the appropriate GPs for checking and exclusion of women (e.g. known to be dead, wrong age, under care for breast cancer, etc.). Owing to updating of the date of birth information at the screening centres after randomisation (mostly for women in the intervention arm), some women are outside the trial age range, but are included in the analyses.

From 1992, randomisation was carried out using software specifically written for the FHSA computer system. Prior to this, randomisation was carried out using numbers generated from the coordinating centre computer for women in three early centres to join the trial. The date of entry is equal to the date of randomisation, except where randomisation was carried out at the coordinating centre; for one of these centres, date of entry was the date when randomisation lists were returned; for the other two, it was set to a date 8 weeks (the mean interval between date of entry and date of first invitation in centres where randomisation was carried out by the FHSA computer system) before the date of first invitation for women in the intervention arm. For women in the control arm, it was set to the date of entry of those in the intervention arm with the same or nearest date of birth in the same general practice.

Between 1993 and 1995, difficulties were encountered in recruiting new centres, due partly to uncertainties over funding and partly to centres being unable to take on additional workload while maintaining NHSBSP screening. After 1996, no further centres were recruited; in 1999, the Data Monitoring Committee recommended that recruitment officially cease, and this was subsequently agreed upon by the Trial Steering Committee. A total of 160 921 women had been randomised, 53 914 to the intervention arm and 107 007 to the control arm; the probability of demonstrating a 20% breast cancer mortality reduction at 10 years of follow-up (under the same assumptions as above) is thus reduced to 73%.

In three centres, Cumbria, Hull and Worthing, screening in the trial ceased prematurely (after four, five and six rounds, respectively) due to inability to manage the additional workload with the available resources. In the primary mortality analysis, women in these centres will be included on an ‘intention-to-treat’ basis, although analyses taking account of the length of intervention may also be considered.

A woman's first trial invitation within 6 months of entry into the study is defined as round one. After the first invitation, a screening round is defined as the offer of a routine trial screen in a 10–14 month period following the previous screen (or invitation to screen). A woman may accrue more than eight screening rounds during the trial, depending on her age in the calendar year of her first invitation. Uptake and outcome of screening are presented by screening rounds, with results combined for later screening rounds as yet incomplete.

An interval cancer is defined as one diagnosed following a negative screen (including return to routine recall following assessment) and occurring before the next offered appointment. Cancers in lapsed attenders are those cancers occurring in women whose last attended screen was negative and who were diagnosed after declining subsequent invitations to attend. Cancers in women who have moved away from their original centre and hence no longer invited are included as ‘lost to screening’.

Recruitment of centres to the trial took place between 1991 and 1996 (Table 1), with randomisation ending in 1997. Results in the present paper include data up to 31st March 2002, with a mean of 8.0 years follow-up from the date of entry. Owing to incomplete ascertainment of breast cancers in the most recent years, incidence rates and the analysis of sensitivity of screening are based on data up to 31st December 1999.

Woman-years for analysis of all-cause mortality are calculated from each woman's date of entry to date of death or 31st March 2002, whichever is earlier. Those for analyses of incidence are calculated from the date of entry to the date of death or 31st December 1999, whichever is earlier, and are also censored at the date of diagnosis of breast cancer.

The sensitivity of screening at different screening rounds has been estimated by the ‘proportional incidence’ method (Day, 1985), using age-specific incidence rates in the control arm to calculate the expected numbers of cancers in the absence of screening. Owing to the fact that the observed incidence in the never-attenders is lower than that in the control arm, the expected incidence *i*_a_ in the attenders has been calculated for each round (assuming everyone is age 40 at first screen, 41 at second screen, etc.) as 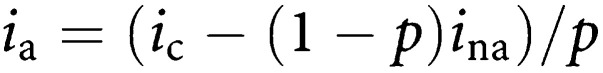
where *p* is the the proportion of ever-attenders, *i*_c_ is the age-specific incidence rate in the control arm (based on a 3-year moving average) and *i*_na_ is the incidence rate in the nonattenders.

Owing to fluctuation due to small numbers, *i*_na_ is estimated as *i*_c_ × *I*_na_/*I*_c_, where *I*_na_ and *I*_c_ are the overall incidence rates in the nonattenders and control arm, respectively.

The primary end point of the trial is a comparison of mortality from breast cancer in the two arms of the trial, in women free from breast cancer at date of entry. All women in the trial are flagged at the National Health Service Central Register (NHSCR), which provides information on deaths from all causes and on cancer incidence. However, a detailed pathology review is also being undertaken (Anderson *et al*, 2002), to enable an earlier analysis based on surrogate outcome measures to be performed (Moss *et al*, 2005).

A radiology review is also being undertaken of all screen-detected, interval and lapsed-attender cancers notified in the trial, in order to identify and highlight the radiological features most frequently missed or misinterpreted when screen reading the mammographic films of younger women, and to identify which features are most helpful to observe when reading mammograms of younger women. The results of this review will be submitted for publication at a later date. During the course of the trial, radiation doses were collected and reviewed; the findings of this work have been published elsewhere (Young, 2002).

‘Contamination’ with private screening in the trial's control population was assessed by postal questionnaires sent to a total of 3755 women in five participating centres, asking about past mammography for both symptomatic and screening purposes. Women were aged 42–49 years at the time of the survey.

## RESULTS

**Figure 1:**
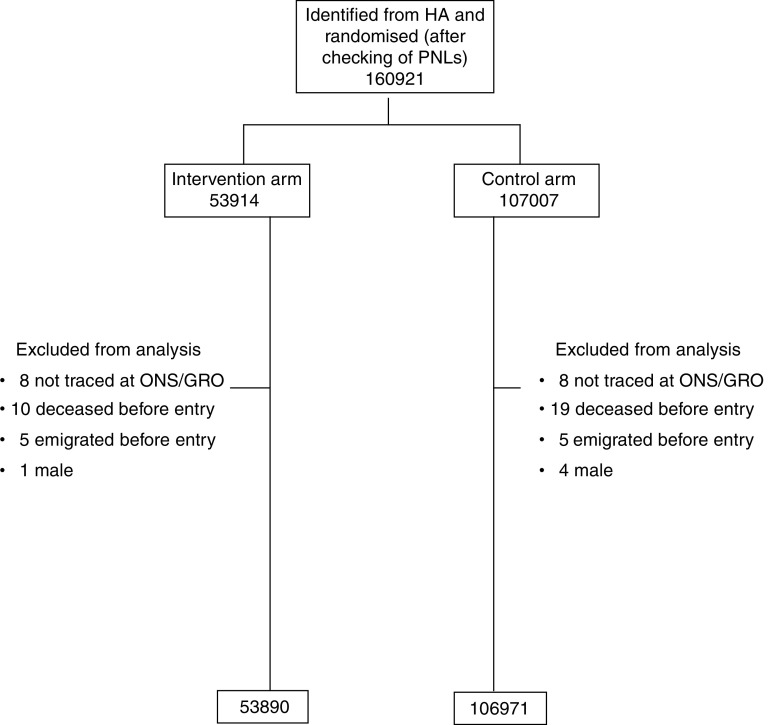
Flow diagram of the progress through the phases of the trial.

Figure 1 and Table 2 show the number of women randomised by trial arm and by individual year of age. A total of 160 921 women were randomised after checking of lists by GP's; more than 99.9% of women randomised have been successfully traced and flagged at NHSCR. A total of 60 women were excluded from analyses for reasons given in the flowchart (Figure 1).

Table 3 shows the uptake of screening at successive rounds, and also demonstrates the loss of women to screening, with 84.3% of women randomised to the intervention arm invited at round five. A total of 1215 women were first invited more than 6 months after their date of entry, and therefore were not invited in round one according to our definition. A further 82 women were never invited due to updates in their Health Authority information, which made them ineligible for invitation (e.g. they were under care or had moved away).

For routine trial screens, 94% of appointments were offered between 10 and 14 months after the previous screen, and 56% after 12 months (±2 weeks). In all, 93% of screens attended took place at 10–14 month intervals.

Data on screening up to and including round five are now complete, but women with the earliest dates of entry will have six or more rounds. Women in the intervention arm have received a mean of 6.6 routine invitations compared with a potential of 7.5 if there had been no loss to follow-up. Uptake of invitations is between 68 and 70% at each round up to round eight, and increases with number of previous attendances; uptake is over 95% in women who have attended at least five previous screens.

In all, 81% of women in the intervention arm have attended at least one invitation for routine screening, and of these, 50% (41% of the intervention arm) have attended all their invitations to routine screening within the trial.

### Screening performance

Table 4 shows the numbers and rates of referrals for assessment, biopsies and *in situ* and invasive cancers detected, by screening round. Mean age will increase with successive rounds, and the rates analysed by age at screening are very similar to those by screening round. Overall rates of referral for assessment are 4.6% at first screen (first invitation) and 3.4% at subsequent screens. Invasive cancer detection rates are 0.09% at first screen, and similar at rescreens until the sixth and later screens. Detection rates of *in situ* cancer (including microinvasive) are 0.02% at first invitation, and increase slightly in the latest rounds. Rates of referral, biopsy and cancer detection are raised in first screens of previous nonresponders.

Table 5 gives the numbers of breast cancers in the control arm and in the intervention arm by method of detection. The incidence rate in women in the intervention arm never attending screening is 1.03 per 1000 women-years, compared with 1.32 per 1000 women-years in the control arm. The incidence rate in the intervention arm is 1.54 per 1000 women-years. A total of 99 invasive interval cancers (plus five *in situ*) have been detected in the 12 months following a negative screen (including return to routine recall following assessment). A further 21 cancers (invasive and *in situ*) have been detected after 12 months but before the woman had received her next invitation. In all, 44 cancers occurred in women who have failed to attend for later screens, and 14 in the ‘lost to screening’ group.

Table 6 shows the numbers of observed and expected invasive interval cancers; the sensitivity of the first screen is 73.6%, and of subsequent screens is 54.1%. For subsequent screens, sensitivity increases from 46.8% at screens two and three to 64.5% at screen six or later.

### All-cause mortality

Table 7 shows the mortality from all causes excluding breast cancer, which is similar in the two arms of the trial (control: 1.29; intervention 1.34 per 1000 women years).

### Screening in the control arm

Of the 3755 women sent questionnaires, 2115 (56.3%) responded and 2041 (54.4%) were suitable for analysis. Of these, 18% reported ever having had a mammogram for symptomatic reasons, and 8.4% reported having had a screening mammogram, 3.9% within the past 3 years.

## DISCUSSION

Despite lower mortality from breast cancer at younger ages, the issue of the effectiveness of screening below age 50 is an important public health question in the UK, due to both demands from the women concerned and the possible implications for the NHS. Although cancer incidence in women in their 40s is approximately half that of women in their 50s, the proportion of all female deaths due to breast cancer is higher in women in their 40s. A third of life-years lost due to breast cancer occurs in women diagnosed with breast cancer in their 40s.

The benefit of mammographic screening was established principally from mortality data on women screened at age 50 or older, and while comparable data in younger women are available from some of the RCTs, none included sufficient numbers to reliably estimate the mortality effect. The most recent overview of the Swedish trials found a relative risk of 0.80 (95% CI 0.63, 1.01) in women aged 40–49 at entry at a median of 15.8 years of follow-up (Nystrom *et al*, 2002). This analysis excluded the Kopparberg arm of the Two County Study. An earlier overview had found a nonsignificant 13% reduction at an average of 9 years of follow-up, the difference emerging after 8 years. A meta-analysis of all RCTs including the Canadian NBSS1 trial found a reduction of 18% in this age group (Hendrick *et al*, 1997). The Canadian NBSS1 trial, which was designed to compare the effect of screening by mammography and physical examination with an initial physical examination only in women aged 40–49 at entry, has suffered from low statistical power, and has also been criticised for the use of a volunteer population and doubts about the quality of mammography. After 11–16 years of follow-up, this trial has shown no difference in breast cancer mortality between the two arms (Miller *et al*, 2002).

Cancer detection rates in the current trial do not increase greatly with age until the later screening rounds at ages 46 or more. Rates of recall for assessment are lower, particularly at the first screen, than for women aged 50–64 in the national programme, but the lower cancer detection rate means that the positive predictive value of recall is lower.

In the early years of the trial, concerns were raised about the uptake of screening that could be achieved in this age group, and about the extent of screening in the control arm, which would dilute any observed effect. In the event, uptake of invitation has remained satisfactory at around 70%, although it appears to fall slightly in the final round(s). The ‘loss to screening’ due to women moving is similar to that which has occurred in other trials, such as the UK Trial of Early Detection of Breast Cancer (Moss *et al*, 1999). Our estimates of the extent of screening in the control arm show little evidence of regular mammography.

The current trial has used annual invitation for screening due to concerns about lower sensitivity of mammography and possible faster mean growth rate of tumours in this age group.

There are limited data available from other studies on cancer detection rates in this age group, particularly at the lower end, and most rates are for women aged 40–49. In the Gothenburg trial, which included five rounds at intervals of 18 months, detection rates in women aged 40–44 at entry were 0.07% at first screen, and 0.18 at subsequent screens.

The trial is set completely within the NHSBSP framework; while this has caused some logistical problems where programmes have had to balance the trial requirements with those of the national programme, it should ensure the applicability of the results to the UK programme.

Uptake in this trial is likely to be lower than would be seen if the national programme were extended to this age group. There has been no health promotion associated with the trial, in order to avoid increasing demand for screening in the control arm. In addition, women who have moved to centres not participating in the trial will not have continued to be routinely invited for screening.

While fieldwork is continuing, there is likely to remain an excess of breast cancers in the intervention arm due to advancement of diagnosis. Screening data collection will continue until all women have been invited for screening as part of the national programme, by which time incidence rates would be expected to be equal in the two arms; this will take until 2009. The trial was designed to compare mortality from breast cancer at 10 years of follow-up. This length of follow-up has now been achieved and a mortality analysis will be conducted once it is decided that data ascertainment is complete. Estimates of mortality reduction based on pathology variables have been made (Moss *et al*, 2005), but conclusions as to the effectiveness and cost-effectiveness of screening in this age group must await definitive results on mortality.

## Figures and Tables

**Table 1:** Recruitment of centres to the trial

**Trial centre**	**Date centre joined study**	**Population contributed**	**Cumulative population**
Guildford	February ‘91	12 110	12 110
Edinburgh	May ‘91	5659	17 769
Derby	June ‘92	10 853	28 622
Avon	August ‘92	8895	37 517
Manchester	September ‘92	7181	44 698
Stoke	September ‘92	8077	52 775
Epping	October ‘92	6938	59 713
N. Nottinghamshire	November ‘92	3621	63 334
Nottingham	November ‘92	8353	71 687
Worthing	January ‘93	6506	78 193
Aylesbury	February ‘93	1942	80 135
Bromley	February ‘93	5834	85 969
Hull	July ‘93	9793	95 762
Swindon	November ‘93	4601	100 363
Bradford	January ‘94	14 425	114 788
Sheffield	July ‘94	6187	120 975
Warrington	September ‘94	7105	128 080
Wirral	October ‘94	7047	135 127
Cumbria	November ‘94	6123	141 250
North London	January ‘95	4455	145 705
Peterborough	February ‘95	4393	150 098
Newcastle	November ‘95	6288	156 386
Swansea	January ‘96	4535	160 921

**Table 2:** Number of women randomised

	**Intervention arm**		**Control arm**	
**Age at entry**	***n***	**%**	***n***	**%**
<38	21	<0.1	8	<0.1
38	158	0.3	285	0.3
39	16 724	31.0	32 841	30.7
40	26 387	49.0	52 141	48.7
41	10 419	19.3	21 597	20.2
>41	181	0.3	99	0.1
				
Total	53 890		106 971	
				
Mean age at entry	40.38		40.39	

**Table 3:** Women invited and uptake of screening (data up to 31 March 2002)

		**Women screened**		
**Screening round**	**Women invited**	***n***	**%**	**% Population screened^a^**
1	52 593	35 846	68	67
2	48 942	33 784	69	63
3	46 985	31 961	68	59
4	45 640	31 311	69	58
5	45 451	31 334	69	58
6	43 030	29 693	69	
7	37 062	26 061	70	Incomplete screening rounds
8	26 832	18 805	70	
⩾9	8923	5917	66	

a Number of intervention arm women screened (by round) expressed as a proportion of the number potentially available for invitation on the basis of the amount of time elapsed since each woman entered the trial.

**Table 4:** Outcome of screening (data up to 31 March 2002)

	**No. screened**	**Referred**	**% of screened**	**Biopsied**	**% of screened**	***In situ* cancer detected**	**% of screened**	**Invasive cancer detected**	**% of screened**	**Total cancer detected**	**% of screened**	**PPV of assessment %**	**PPV of biopsy %**
First invitation	35 846	1655	4.6	93	0.26	6^a^	0.02	31	0.09	37	0.10	2	40
First screening round in previous nonresponders	7679	511	6.7	48	0.63	3	0.04	14	0.18	17	0.22	3	35
Second routine screening round	39 912	1324	3.3	70	0.18	8	0.02	33	0.08	41	0.10	3	59
Third routine screening round	37 128	1323	3.6	77	0.21	8	0.02	34	0.09	42	0.11	3	55
Fourth routine screening round	34 082	1161	3.4	75	0.22	9^a^	0.03	28	0.08	37	0.11	3	49
Fifth routine screening round	29 818	989	3.3	61	0.20	12^b^	0.04	23	0.08	37^c^	0.12	4	61
Subsequent routine screening rounds	60 206	2072	3.4	166	0.28	28^d^	0.05	69	0.11	98^e^	0.16	5	59

PPV=positive predictive value.

a This value includes one microinvasive case.

b This value includes four microinvasive cases.

c This value includes two cases where invasive status is not known.

d This value includes two microinvasive cases.

e This value includes one case where invasive status is not known.

**Table 5:** Breast cancers by method of detection (data up to 31 December 1999)

	**Intervention arm**	**Control arm**
Between randomisation and invitation	9	
Never-attenders	61	
Screen detected: total	229	
		
*Interval cancers*		
Before next invitation	125	
Lapsed attenders	44	
‘Lost to screening’	14	
		
Total	482	821

*In situ* and microinvasive cases included.

**Table 6:** Interval cancers and estimate of sensitivity (data up to 31 December 1999)

	**Interval cancers within 12 months (*O*)**	**Expected cancers (*E*)**	**Sensitivity^a^ (%)**
1st screen	13	49.4	73.6
2nd and 3rd screen	47	88.4	46.8
4th and 5th screen	28	68.1	58.9
Later screens	11	31.0	64.5

*In situ* and microinvasive cases excluded.

a (1−*O*/*E*) × 100%.

**Table 7:** All-cause mortality excluding breast cancer, by year in trial (data up to 31 March 2002)

	**Control arm**				**Intervention arm**				**Total**			
**Year in trial**	**No. of women**	**Woman-years**	**Deaths**	**Rate per 1000 w years**	**No. of women**	**Woman-years**	**Deaths**	**Rate per 1000 w years**	**No. of women**	**Woman-years**	**Deaths**	**Rate per 1000 w years**
1	106 971	106 908	86	0.80	53 890	53 862	42	0.78	160 861	160 770	128	0.80
2	106 871	106 824	107	1.00	53 847	53 822	61	1.13	160 718	160 646	168	1.05
3	106 746	106 669	121	1.13	53 777	53 739	61	1.14	160 523	160 408	182	1.13
4	106 609	106 538	137	1.29	53 711	53 669	82	1.53	160 320	160 207	219	1.37
5	106 449	106 346	138	1.30	53 623	53 574	73	1.36	160 072	159 920	211	1.32
6	106 276	104 648	152	1.45	53 533	52 698	79	1.50	159 809	157 346	231	1.47
7	100 386	92 599	137	1.48	50 539	46 660	68	1.46	150 925	139 259	205	1.47
8	84 076	69 840	116	1.66	42 398	35 188	62	1.76	126 474	105 028	178	1.69
9	54 314	42 284	77	1.82	27 326	21 252	36	1.69	81 640	63 536	113	1.78
10	30 375	16 539	42	2.54	15 278	8343	15	1.80	45 653	24 882	57	2.29
11	7470	4245	4	0.94	3739	2011	5	2.49	11 209	6256	9	1.44
												
Total		863 440	1117	1.29		434 818	584	1.34		1 298 258	1701	1.31
